# Medical Staffs of Voluntary Hospitals

**Published:** 1924-08

**Authors:** C. E. Stewart Flemming


### MEDICAL STAFFS OF VOLUNTARY HOSPITALS.

### SHOULD THEY BE PAID?

By C. E. STEWART-FLEMMING, M.R.C.S., L.R.C.P.

The decrease of charitable contributions and the greatly increased cost of the whole conduct of hospitals has compelled managers to require payment, directly or indirectly, from the great majority of patients. It is to the credit of the working-man of to-day that he has that spirit of independence which makes him unwilling to receive this benefit as a charity. He resents the inquiries of the almoner or any suggestion of charity when he considers the large payments made by his class, to which he himself has contributed on the assumption that he could claim admission and treatment as a right. And it is these contributory schemes in one form or another, together with the direct payment required from or on behalf of patients, that have of late years kept the hospitals alive. It is obvious that these methods of payment must continue in increasing proportion to provide hospital income.

### An Inevitable Change.

What is practically a system of contract practice is being introduced. It is inevitable; and the position of the medical staff under this new order of voluntary hospitals must be fundamentally affected. The generally accepted definition of a voluntary hospital is in essence its free and independent management, and that independence of management is largely conditional on the voluntary nature of the income. This definition does not in any way imply the existence of gratuitous medical service. The Cave Committee in their Report say that the payment of the staff beyond what is really an arbitrary point would endanger the future of the voluntary system, but we must remember that in two at least of the largest London hospitals the staff receive a regular and fixed honorarium. If payment for medical service is, as we are told, to "knock the bottom out of the voluntary system," then the hospitals themselves must have already accomplished the task by requiring and accepting payment from their patients. However, none of them has in consequence ceased to call itself a voluntary institution. In 1912, 20 per cent. of the income of the voluntary hospitals was derived from patients' payments, workmen's collections, etc. In 1922 these produced over 47 per cent. of the income.

### The New Circumstances.

This change, even in its beginning, requires the recognition of the need for compensating change in the conditions of the service of the medical staff. If this is not done early it will be far more difficult to do it when the altered system has become firmly established. The medical profession is as anxious to-day as ever it was to do philanthropic work, but necessity born of altered circumstances compels it to reconsider its position. Many who formerly were unable to contribute toward the cost of their hospital treatment are now, through mutual benefit contributory schemes and other like methods, able to pay a large part of that cost; and, on the other hand, a large proportion of those who formerly obtained treatment in their own homes or in nursing homes are now unable to do so, partly because their incomes are much smaller and partly because modern methods of treatment have become so much more elaborate and costly, and because this treatment can only be provided effectively in a well-equipped institution.

### The Need for Efficiency.

It can hardly be necessary to point out that a medical staff to carry out the exacting work of a modern hospital must not only be ample in size and have a good supply of recruits, but that its members must be the most efficient that can be found. They must be willing and able to give freely their time and thought, and to do this they must feel that the time they are devoting to their hospital work is not preventing them from earning the means to keep themselves and their families in some sort of comfort, and that they have not to spend all their time outside the hospital in a tiring endeavour to make up for the hours they have spent in it, because for the proper conduct of their work in the wards they will require to give a very considerable time to reading, research, scientific meetings and discussions. Senior members find it increasingly difficult as years go on to give the necessary hours to their hospital work, and younger members often can hardly make ends meet if they do not devote enough time to that sort of private work which provides so small a return that it takes nearly all the day to make a living. The result is that it is becoming increasingly difficult to find young men to take up this work unless they have some assistance. More men than ever are required, and they will actually have to come from the ranks of those who in the past made their living by attending those very patients who are now resorting to the hospitals for treatment. It is only by throwing open more hospital appointments to young men, making the term of office shorter, and offering some payment for the work, that it will, under the altered circumstances of these days, be possible to attract to the service, to select and to keep there the keenest and ablest men in the profession. What is now happening only too often is that the keen young man takes up the more easily obtained contract and private practice, and that the work of many an enthusiast is lost to the hospital.

### A Form of Hospital Abuse.

It is in consideration of the large new class of patients that are using the hospitals, of the altered conditions of payment to the hospitals by the great majority of patients, of the great amount of work done for the general benefit of the community, that the members of the medical staffs feel that they have a just claim for some remuneration for time and service given. They do not desire what could be considered full payment—that would not be practicable. Some years ago Sir Henry Burdett asked: "Are committees of large hospitals justified in continuing to administer so great an amount of free medical relief as they do at present?" The accepting of treatment at hospitals by those who can afford to pay for it, without any remuneration being given to the medical staff, does in fact constitute a form of hospital abuse, and the demand for payment actually began after this abuse started. It first took effect when the treatment of war pensioners was undertaken, and it became obvious that the medical staff should properly receive a share of the money paid by the Ministry for these patients. The increasing intervention of the State in the treatment of disease in other patients forced more general attention to the question, and when the hospitals themselves were compelled by circumstances to require payment from or on behalf of the majority of their patients, the profession naturally felt that these altered circumstances justified them in reconsidering their position in regard to the hospitals.

### The "Staff Fund."

It is not a proper argument to say that medical men will of course continue to treat gratuitously members of the working classes who contribute to the hospitals through some provident scheme, because they treated these same patients in the past gratuitously. Formerly this class could not afford to pay for many things that they now do; also they had not any provident system by which their own treatment could be paid for when necessary. As the Labour Party say in their memorandum: "If a hospital accepts the responsibility for the maintenance and treatment of a certain class of patients in return for payment, the profession would be right in staking out its claim for remuneration." A point generally overlooked is how much medical men contribute to that hospital revenue which is derived from patients' payments. It is through their work and theirs alone that the hospital is entitled to make any charge. If the principle of payment is accepted, the form in which it is made is not for the moment so important. Probably an honorarium would be the most convenient and in some ways the most acceptable, but there are many who think that by allocating to a special fund, to be called "The Staff Fund," a percentage of all payments made by or on behalf of patients, it might be possible to remunerate the staff for those patients only who were not strictly speaking the objects of charity, leaving them free to treat gratuitously, as they have always done, the destitute poor. This method would be an approach to the system adopted in general practice—of regulating charges in some measure according to the financial circumstances of the patient. This is in fact a fair and not ungenerous arrangement.

### The Example of the Hospitals.

It is frequently urged that the patients' payments, whether direct or indirect, should only be assessed for Staff Fund purposes when the payment made is in excess of that required for maintenance, but there is little doubt that members of a contributory scheme or individual patients regard the money they give as being for all hospital services, including treatment, and not for maintenance alone. In fact, it is to obtain this treatment that they contribute; maintenance and accommodation are only regarded—if thought of at all—as a natural adjunct. Much objection has been taken to the suggestion that such a Staff Fund should be even in part built up by payments from contributions from the poor who have just managed to scrape together for the hospital a few shillings, but no objection is taken to the action of the hospital itself in exacting from the poor these few shillings. The hospitals, quite rightly, require the patients to subscribe as much as they can afford.

### Not Charity but Business.

The working classes do not want charity; they want to be treated as a business proposition. When they contribute to a hospital fund, however small the amount, they do so believing that they are paying for the services of the doctor. Subscribers to a provident scheme are hardly likely to object to the payment of the staff, and is not the fear that this movement may adversely affect public support of our voluntary hospitals rather an imaginary one? There has been no sign of resentment at the hospitals themselves doing exactly what the medical men want to do.

### The "Valuable Appointment" Argument.

Perhaps the most serious argument brought against the payment of medical staffs is that their appointments are valuable and give opportunity for experience and fame hardly to be obtained in any other way, and bring in indirectly considerable income. This view applies chiefly if not only to hospitals in large centres, where positions on the staff are valuable and indirectly lucrative to a certain number of the holders. The advantages, repute and profit said to attach to these appointments are much exaggerated; the value is potential and depends on the personality of the holder. The appointment is indeed his opportunity; he might use his chance either for unremunerative scientific research or for professional work on behalf of the patients in the hospital which may lead to lucrative practice. In the case of the smaller hospitals there are no gates open only to a select few and leading to these "lucrative fields of practice." Every practitioner working there cultivates a crop for his own consumption; there is no outside market for it. The work he does there brings him little or no reputation outside the the district in which he practises.

### Doing Away with the Need for Charity.

There are many medical men not happy in that they have a feeling that this movement may be interfering with the cause of charity of which they have always been champions; but there is something better—that is, to do away with the need for charity. The need is diminishing, and just so far as it is there is greater need for hospitals for those who can afford to pay. We must consider the point of view of those who cling to old ideals, but they must remember that by so doing they may endanger the future.

---

**Extension of Princess Alice Orphanage.**

The extension of the Princess Alice Orphanage, the Birmingham Branch of the National Children's Home, includes a new hospital, given and equipped by Mr. and Mrs. G. E. Lowe, of Sutton Coldfield, which has now been opened. The hospital possesses two main wards, small isolation wards, a surgery and dispensary, staff rooms and kitchen.

